# Evaluation of *Chlorella* as a Decorporation Agent to Enhance the Elimination of Radioactive Strontium from Body

**DOI:** 10.1371/journal.pone.0148080

**Published:** 2016-02-01

**Authors:** Kazuma Ogawa, Tadahisa Fukuda, Jaegab Han, Yoji Kitamura, Kazuhiro Shiba, Akira Odani

**Affiliations:** 1 Graduate School of Medical Sciences, Kanazawa University, Kanazawa, Japan; 2 Institute for Frontier Science Initiative, Kanazawa University, Kanazawa, Japan; 3 DAESANG Corporation, Seoul, Korea; 4 Advanced Science Research Center, Kanazawa University, Kanazawa, Japan; ENEA, ITALY

## Abstract

**Background:**

Release of radionuclides, such as ^137^Cs and ^90^Sr, into the atmosphere and the ocean presents an important problem because internal exposure to ^137^Cs and ^90^Sr could be very harmful to humans. *Chlorella* has been reported to be effective in enhancing the excretion of heavy metals; thus, we hypothesized that *Chlorella* could also enhance the elimination of ^137^Cs or ^90^Sr from the body. We evaluated the potential of *Chlorella* as a decorporation agent *in vitro* and *in vivo*, using ^85^Sr instead of ^90^Sr.

**Methods:**

*In vitro* experiments of adsorption of ^137^Cs and ^85^Sr to *Chlorella* were performed under wide pH conditions. The maximum sorption capacity of *Chlorella* to strontium was estimated using the Langmuir model. A ^85^Sr solution was orally administrated to mice pretreated with *Chlorella*. At 48 h after ^85^Sr administration, the biodistribution of radioactivity was determined.

**Results:**

In the *in vitro* experiments, although ^85^Sr barely adsorbed to *Chlorella* at low pH, the ^85^Sr adsorption ratio to *Chlorella* increased with increasing pH. The maximum sorption capacity of *Chlorella* to strontium was 9.06 mg / g. ^137^Cs barely adsorbed to *Chlorella* under any pH conditions. In the biodistribution experiments, bone accumulation of radioactivity after ^85^Sr administration was significantly decreased in the *Chlorella* pretreatment group compared with the non-treatment control group.

**Conclusions:**

In conclusion, these results indicated that *Chlorella* could inhibit the absorption of ^90^Sr into the blood and enhance the elimination of ^90^Sr from the body through adsorption in intestine. Further studies are required to elucidate the mechanism and the components of *Chlorella* needed for adsorption to strontium and could promote the development of more effective decorporation agents.

## Introduction

Radionuclides released into the atmosphere and the ocean by nuclear power plant accidents, such as the Chernobyl and Fukushima disasters, are important problems for the ecosystems and humans [1–5]. Among the released radionuclides, cesium-137 (^137^Cs) and strontium-90 (^90^Sr) are considered to be harmful to humans because both have a long half-life and can contaminate aquatic ecosystems and food products due to their high solubility in water [3].

^137^Cs (t_1/2_ = 30.1 y) is one of the most important nuclear fission elements and decays to barium-137m (^137m^Ba) with the emission of β-particles (0.51 MeV). ^137m^Ba in turn decays to the stable isotope ^137^Ba with the emission of gamma rays (0.66 MeV) by isomeric transition. Because the physical half-life of ^137m^Ba (t_1/2_ = 2.55 m) as a daughter radionuclide is sufficiently short compared with that of ^137^Cs, a radioactive equilibrium is established. Cesium is an alkaline metal and a member of the IA family of the periodic table. As its chemical properties are similar to those of potassium, cesium is immediately absorbed after intake and is fairly uniformly distributed throughout body [6]. In particular, cesium shows high accumulation in muscle and is retained in the entire body, with a biological half-life of approximately 100 days in healthy adult male humans [7].

^90^Sr (t_1/2_ = 29.1 y) is another important nuclear fission radionuclide. ^90^Sr decays to yttrium-90 (^90^Y) with the emission of β-particles (0.54 MeV). ^90^Y in turn decays to the stable isotope zirconium-90 (^90^Zr) with the emission of high energy β-particles (2.27 MeV). Because the physical half-life of ^90^Y (t_1/2_ = 2.67 d) as a daughter radionuclide is sufficiently short compared with that of the parent radionuclide ^90^Sr, a radioactive equilibrium is established. Strontium (Sr) is an alkaline earth metal and a member of the IIA family of the periodic table. Therefore, strontium acts as a calcium mimic and shows high accumulation in bone through incorporation into mineralizing collagen during new bone formation [8,9]. The biological half-life of ^90^Sr is very long [10]. Therefore, internal exposure to ^90^Sr is associated with the development of leukemia and osteosarcoma [11,12].

Because of the properties mentioned above, internal exposure to ^137^Cs and ^90^Sr radionuclides can be very harmful to humans. Compounds that inhibit the absorption of these radionuclides from the gastrointestinal tract into the blood and enhance their elimination after intake are useful in decreasing the absorbed radiation dose when people are exposed to these radionuclides. Indeed, insoluble ferric(III) hexacyanoferrate (Fe_4_^III^[Fe^II^(CN)_6_]_3_)·xH_2_O (Radiogardase^®^, Prussian blue insoluble, PB), a compound that promotes the elimination of ^137^Cs from the body, is useful for the treatment of radiocesium poisoning and has been approved for use by regulatory bodies, such as U.S. Food and Drug Administration (FDA) and European Medicines Agency (EMA). In the case of ^90^Sr, some compounds such as alginate promote excretion as reported from basic research [13,14].

*Chlorella* is a genus of single-cell green algae that grows in fresh water. It is known as a health food composed of approximately 1%–4% chlorophyll, 55%–67% protein, 9%–18% dietary fiber, and large amounts of minerals and vitamins [15]. In a previous study, *Chlorella* was shown to enhance the excretion of cadmium (Cd) by inhibiting intestinal Cd absorption in rats [16]. In another report, lead (Pb) concentration in blood was markedly reduced when Pb and *Chlorella vulgaris* extract were simultaneously administrated compared with when only Pb was administrated [17]. Furthermore, another study reported enhancement of mercury (Hg) elimination by *Chlorella* [18]. Because *Chlorella* has been shown to be effective in enhancing the excretion of heavy metals, we hypothesized that *Chlorella* could also inhibit the absorption and enhance the elimination of radioactive cesium or strontium from the body after their oral intake. We evaluated the potential of *Chlorella* as a decorporation agent for radioactive cesium and strontium *in vitro* and *in vivo*. In this study, ^85^Sr (T_1/2_ = 64.8 d) was used instead of ^90^Sr because ^85^Sr emits gamma rays that can be measured easily.

## Materials and Methods

### Materials

^85^SrCl_2_ (> 111 GBq/g) was purchased from PerkinElmer (Waltham, MA, USA). ^137^CsCl was purchased from Eckert & Ziegler Isotope Products Inc. (Valencia, CA, USA). Chlorella powder was supplied by Daesang Corp. (Seoul, Korea). Other reagents were of reagent grade and used as received.

### *In vitro* experiments of adsorption of ^137^Cs and ^85^Sr to *Chlorella*

*Chlorella* (10, 30, or 100 mg) was suspended and ^137^Cs or ^85^Sr was added in 1 mL of the first test solution (artificial gastric juice, pH 1.2) or the second test solution (artificial intestinal juice, pH 6.8) defined in the Japanese Pharmacopoeia. After shaking the suspension at 1,000 rpm and 37°C for 1 h using a shaking incubator (SI-300C; AS ONE Corp., Osaka, Japan), the samples were centrifuged at 10,000*g* and room temperature for 10 min. The radioactivity of the supernatant was measured using an auto-well gamma counter (ARC-380; Hitachi Aloka Medical, Ltd., Tokyo, Japan), and the counts were corrected for background radiation. Control experiments were performed using the same procedure but without *Chlorella*. The binding ratios were determined as follows:

Binding ratio to *Chlorella* (%) = [1 − (radioactivity of the supernatant of each sample) / (radioactivity of the supernatant of the respective control)] × 100

### pH dependence of *in vitro* adsorption of ^137^Cs and ^85^Sr to *Chlorella*

*Chlorella* (30 mg) was suspended and ^137^Cs or ^85^Sr was added in 1 mL of 0.01 M HEPES buffer solution (pH 2–13), and shaking, centrifugation, and radioactivity measurement were conducted as described above. The pH of each suspension after shaking was measured.

Reversibility of the adsorption potential between *Chlorella* and ^85^Sr with pH variation was evaluated. Chlorella (30 mg) was suspended in 1 mL of the first test solution (pH 1.2) defined in the Japanese Pharmacopoeia and then shaken at 1,000 rpm and 37°C for 1 h. After centrifugation at 10,000*g* and room temperature for 10 min, 800 μL of the supernatant was removed. Following this, 23, 25, or 27 μL of 1M NaOH solution and 777, 775, or 773 μL of 0.01M HEPES buffer solution (pH 8) were added to the *Chlorella* suspension. Thereafter, ^85^Sr was added to the Chlorella suspension, and the suspension was shaken at 1,000 rpm and 37°C for 1 h. After centrifugation at 10,000*g* and room temperature for 10 min, the radioactivity and pH of the supernatant were measured as described above.

### Langmuir model

*Chlorella* suspension samples (10 mg/mL) containing 1, 10, 50, 100, 200, 500, and 1000 ppm of Sr and 832.5 kBq/mL of ^85^Sr as a final concentration in 0.02 M HEPES buffer (pH 7.4) were prepared by mixing radioactive strontium and non-radioactive strontium. After shaking at 1,000 rpm and 37°C for 1 h, the binding ratio of each sample to *Chlorella* was determined using the method described above.

### Effects of cations (Na^+^, K^+^, or Ca^2+^) on *in vitro* adsorption of ^85^Sr to *Chlorella*

*Chlorella* (10 mg) was suspended and ^85^Sr was added in 1 mL of 0.02 M HEPES buffer solution containing 500, 1000, or 10000 ppm of Na^+^, K^+^, or Ca^2+^, which were prepared by dissolution of NaCl, KCl, or CaCl_2_. After shaking at 1,000 rpm and 37°C for 1 h, the binding ratio of each sample to *Chlorella* was determined using the method described above.

### *In vivo Chlorella* experiments for promoting excretion of ^85^Sr from the body

Experiments with animals were conducted in strict accordance with the Guidelines for the Care and Use of Laboratory Animals of Kanazawa University. The animal experimental protocols used were approved by the Committee on Animal Experimentation of Kanazawa University (Permit Number: AP-143039). The animals were housed with free access to food and water at 23°C with a 12-hour alternating light/dark schedule. *Chlorella* suspended in a 5% aqueous glucose solution (75 mg / 500 μL) was orally administrated to 6-week-old male ddY mice (n = 19, 27–30 g, Japan SLC, Inc., Hamamatsu, Japan) 5 times every 90 min. The mice were fasted for 36 h after the first administration of *Chlorella*. Thirty minutes after the final administration of the *Chlorella* suspension, a ^85^Sr solution (24–40 kBq / 100 μL) was orally administrated to the mice. In the control group (n = 28), 500 μL of a 5% aqueous glucose solution was administrated instead of the *Chlorella* suspension. In the alginate group (n = 14), sodium alginate in a 5% aqueous glucose solution (15 mg / 500 μL) was administrated instead of the *Chlorella* suspension. Mice were sacrificed 48 h after ^85^Sr administration. Tissues of interest were removed and weighed, and radioactivity counts were measured. Complete left femurs were isolated as representative bone samples. Whole-body in Table 1 means the sum of whole-body radioactivity except the gastrointestinal tract.

**Table 1:** Biodistribution of radioactivity at 48 h after oral administration of ^85^Sr in mice with pretreatment of *Chlorella* or alginate.

Tissue	Control	Chlorella	Alginate
Blood	0.02 (0.01)	0.02 (0.02)	0.02 (0.01)
Liver	0.01 (0.00)	0.01 (0.00)	0.01 (0.00)
Kidney	0.03 (0.01)	0.02 (0.01)	0.02 (0.01)
Small-intestine^*^	0.09 (0.04)	0.08 (0.03)	0.24 (0.23)^†^
Large-intestine^*^	0.16 (0.07)	0.14 (0.07)	0.50 (0.47)^†^
Spleen	0.05 (0.06)	0.02 (0.04)	0.01 (0.01)^†^
Pancreas	0.02 (0.02)	0.02 (0.02)	0.02 (0.01)
Lung	0.03 (0.01)	0.03 (0.03)	0.02 (0.01)
Heart	0.02 (0.02)	0.02 (0.03)	0.01 (0.01)
Stomach^*^	0.04 (0.02)	0.03 (0.02)	0.73 (0.91)^†^
Bone	29.28 (5.15)	25.96 (4.32)^†^	27.79 (4.08)
Muscle	0.03 (0.04)	0.02 (0.03)	0.01 (0.01)
Whole-body^*^	26.83 (4.60)	23.14 (3.22)^†^	28.58 (4.88)

Expressed as % injected dose per gram. Each value represents the mean (SD) for twenty-eight (control), nineteen (*Chlorella*), or fourteen (alginate) animals.

^*^Expressed as % injected dose.

^†^*p* < 0.05 vs. Control

### Statistical evaluation

Data are expressed as means ± standard deviations where appropriate. Dunnett’s multiple comparison test compared with the control group was used for *in vivo* experiments. Results were considered statistically significant at *p* < 0.05.

## Results

### *In vitro* experiments of adsorption of ^137^Cs and ^85^Sr to *Chlorella*

**Fig 1:**
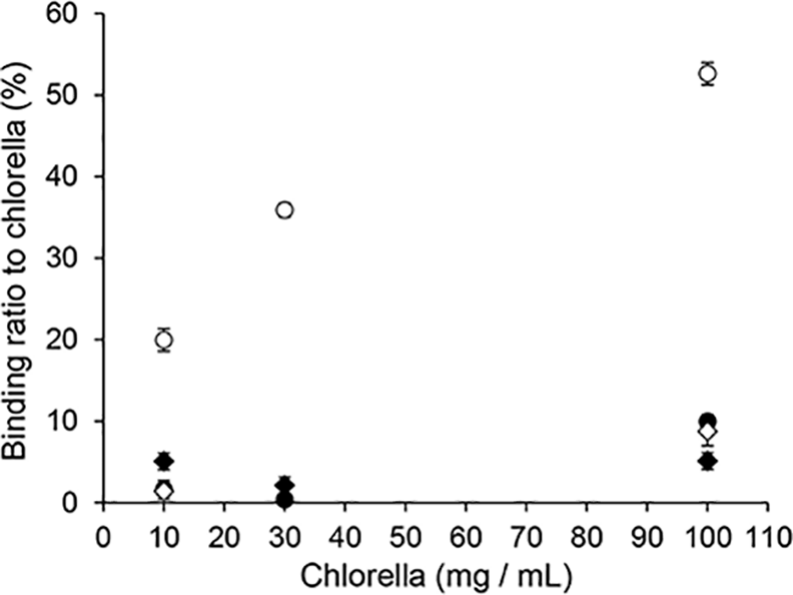
Adsorption of radionuclides to *Chlorella*. Binding ratios of ^85^Sr at pH 1.2 (closed circles), ^85^Sr at pH 6.8 (open circles), ^137^Cs at pH 1.2 (closed diamonds), or ^137^Cs at pH 6.8 (open diamonds) to *Chlorella* depended on the *Chlorella* concentration. Data are expressed as the mean ± SD of three samples.

The adsorption ratios of ^137^Cs and ^85^Sr to *Chlorella* are shown in Fig 1. ^85^Sr adsorbed to *Chlorella* in a quantity-dependent manner under a neutral pH condition. However, it barely adsorbed to *Chlorella* under an acidic pH condition. ^137^Cs barely adsorbed to *Chlorella* under both neutral and acidic pH conditions.

### pH dependence of *in vitro* adsorption of ^85^Sr to *Chlorella*

**Fig 2:**
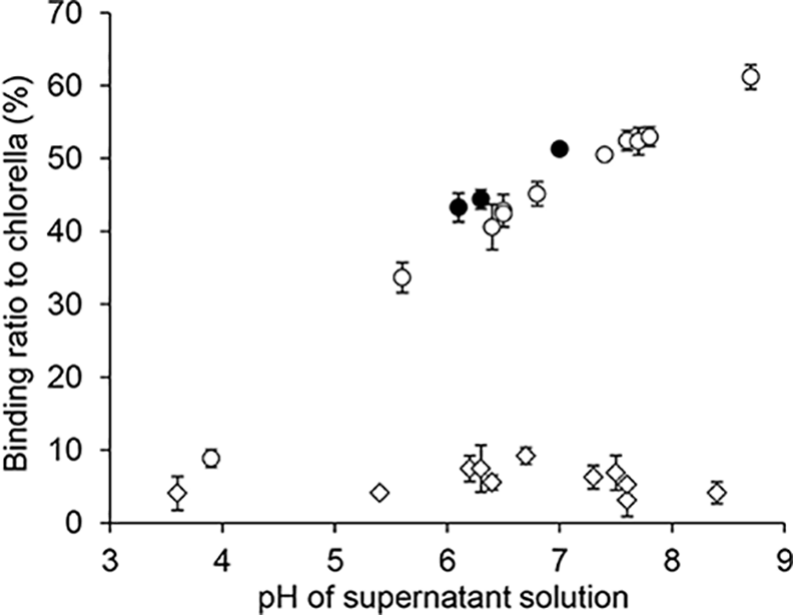
pH dependence of *Chlorella* adsorption. Binding ratios of ^85^Sr (open circles) or ^137^Cs (open diamonds) to *Chlorella* depended on the pH conditions. Binding ratios of ^85^Sr (closed circles) after exposure to acidic conditions. Data are expressed as the mean ± SD of three samples.

The open circles in Fig 2 show the pH dependence of ^85^Sr adsorption to *Chlorella*. Although ^85^Sr barely adsorbed to *Chlorella* at low pH, its adsorption ratio to *Chlorella* increased with increasing pH. The closed circles in Fig 2 show ^85^Sr adsorption to *Chlorella* at pH 6–7 after exposure to acidic conditions. It was confirmed that ^137^Cs was barely adsorbed to *Chlorella* under any pH conditions (open diamonds).

### Langmuir model

Adsorption capacity of Chlorella with Sr was evaluated by the Langmuir model, defined as follows:

$$q=\frac{aq_{\text{max}}\;C_{\text{eq}}}{1+a\;C_{\text{eq}}}$$ (1)

where *q* (mg / g) represents Sr binding per Chlorella, *C*_eq_ (mg / L) is the equilibrium concentration of strontium, *q*_max_ (mg / g) is the maximum sorption capacity, and *a* (L / mg) is the sorption constant.

**Fig 3:**
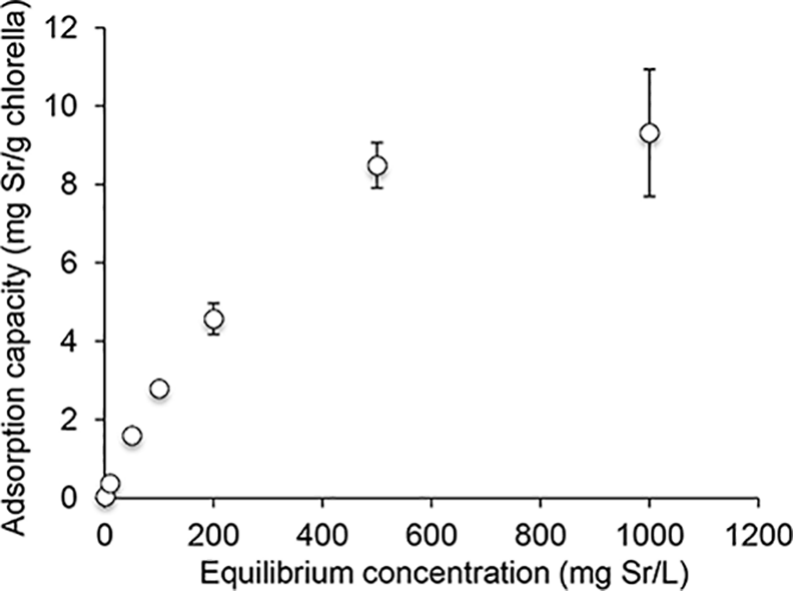
Langmuir model. Adsorption capacity of *Chlorella* to strontium. Data are expressed as the mean ± SD of three samples.

Fig 3 shows that the binding of strontium to *Chlorella* increased at low metal concentrations and began to level off at high concentrations, indicating saturation of the adsorption sites. These data showed an excellent fit with the Langmuir model, indicating the correct assumption of a uniform surface with finite identical sites and monolayer adsorption of the adsorbate. The maximum sorption capacity was estimated to be 9.06 mg / g.

### Effects of cations (Na^+^, K^+^, or Ca^2+^) on *in vitro* adsorption of ^85^Sr to *Chlorella*

**Fig 4:**
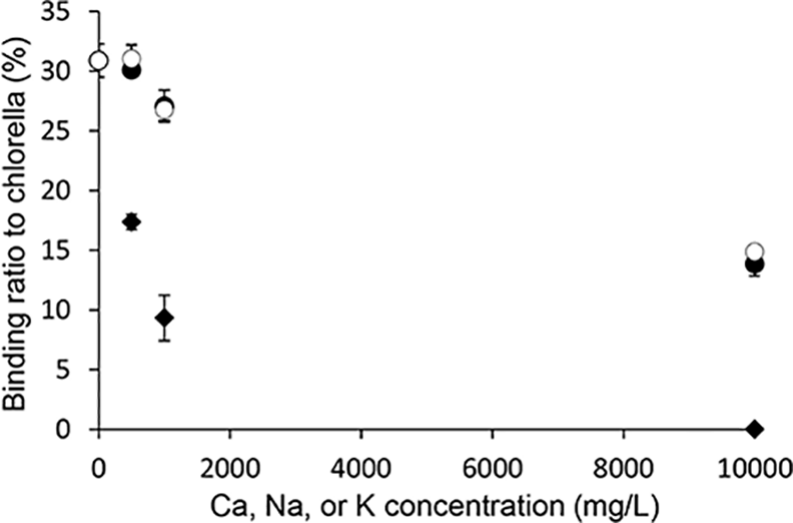
Adsorption of ^85^Sr to *Chlorella* in the presence of cations. Binding ratios of ^85^Sr to *Chlorella* in the presence of Na^+^ (closed circles), K^+^ (open circles), or Ca^2+^ (closed diamonds). Data are expressed as the mean ± SD of three samples.

Fig 4 shows that the binding of strontium to *Chlorella* was decreased by the presence of cations. Although Na^+^ and K^+^ hardly decreased the binding of strontium to *Chlorella* at low concentration of the cations, but the addition of Ca^2+^ remarkably decreased the binding of strontium to *Chlorella* even at low concentration of Ca^2+^.

### *In vivo* experiments for promoting excretion of ^85^Sr from the body

Table 1 lists the biodistribution of radioactivity at 48 h after oral administration of ^85^Sr with pretreatment of *Chlorella* or sodium alginate, or without pretreatment as a control group. Pretreatment with *Chlorella* significantly decreased the accumulation of radioactivity in the bone and the whole-body (excluding the gastrointestinal tract) compared to the control group. Meanwhile, the difference between radioactivity in bone and whole body was not statistically significant between control group and sodium alginate treated group. Little radioactivity existed in almost all tissues except the bone.

## Discussion

In *in vitro* adsorption experiments, *Chlorella* did not adsorb ^137^Cs at any pH range or concentration. Therefore, *in vivo* experiments were not performed using ^137^Cs. On the other hand, we had anticipated that *Chlorella* could adsorb radioactive strontium because a column packed with *Chlorella vulgaris* had previously been proposed as a means to remove strontium cations from contaminated aqueous solutions [19]. ^85^Sr adsorbed to *Chlorella* under neutral and weak basic pH conditions, whereas it barely adsorbed under acidic pH conditions. The low pH of the gastric juice must prevent the adsorption of ^85^Sr to *Chlorella*. Therefore, any inhibition of absorption or enhanced excretion of radiostrontium observed *in vivo* must have been the result of adsorption of strontium to *Chlorella* in the intestine. Thus, we investigated whether the *Chlorella* sample that was exposed to an acidic condition and then adjusted to neutral pH could adsorb ^85^Sr or not. The results indicated that after exposure to an acidic solution, *Chlorella* still adsorbed ^85^Sr to the same degree at neutral pH compared with the samples not exposed to the acidic solution (Fig 2). These results indicated that *Chlorella* could bind radiostrontium in the intestine after passing through the acidic conditions in the stomach.

From the results of *in vitro* experiments, it was assumed that abundant *Chlorella* in the intestine could adsorb radioactive strontium and inhibit absorption, resulting in enhanced excretion of radioactive strontium. Thus, *in vivo* experiments to enhance the elimination of radiostrontium were designed using mice given multiple oral administrations of *Chlorella*. The results of the *in vivo* experiments showed that the accumulation of radioactivity in the bones and the whole-body except the gastrointestinal tract in the *Chlorella* treatment group was significantly lower than that in the control group. As described in the Introduction section, it has been known that orally administrated strontium accumulates at high levels in the bone after absorption into the blood. The bone accumulation of radioactivity after intake of radioactive Sr must be correlated to AUC of radioactivity level in blood (time-activity curve) because Sr must not be metabolized and Sr is not released from bone after adsorption [20,21]. Therefore, the bone accumulation could be index of the radioactivity level of Sr in blood. The difference in the biodistribution after ^85^Sr administration between the *Chlorella* treatment group and the control group indicates that *Chlorella* inhibited ^85^Sr absorption into blood and enhanced its excretion as a result of adsorption between *Chlorella* and ^85^Sr in the intestine.

It has been reported that alginate reduces absorption and enhances excretion of strontium [22–24], but the alginate did not inhibit absorption of ^85^Sr in *in vivo* experiments of this study because the bone accumulation of radioactivity in the alginate treated group was not significantly reduced compared to that in the control group. Although the reason for this is not known, in a previous report using rats, whole-body retention of ^85^Sr was not significantly changed by treatment of single oral administration of alginate (2%, 1 mL) just before oral administration of ^85^Sr. Meanwhile, when ^85^Sr was orally administrated after feeding on 10% alginate containing diet for 10 days, the whole-body retention of ^85^Sr in alginate-treated rats was decreased sharply compared with that in control rats [23]. Thus, preadministration for a longer period of time or preadministration of a larger amount alginate might be necessary to enhance excretion of ^85^Sr in this study.

As mentioned above, ^85^Sr absorption was significantly inhibited in the *Chlorella* treatment group compared with the control group, but the inhibitory effects of *Chlorella* were smaller than expected. This finding seems to be derived from some of following causes. First, it has been reported that *Chlorella* promotes dioxin excretion [15]. As some of the dioxin that accumulates in the body is secreted with bile into the intestine [25], *Chlorella* could inhibit not only dioxin absorption but also dioxin reabsorption in the intestine, thereby promoting excretion of dioxin from the body into the feces. On the other hand, strontium barely enters the enterohepatic circulation. It is known that after absorption into blood, strontium mainly accumulates in the bones or is mainly excreted into the urine via kidney. Therefore, the strategy of using *Chlorella* as a decorporation agent for radioactive strontium can be applied only before absorption; thus, *Chlorella* has a lower chance of encountering strontium compared with other target compounds that enter the enterohepatic circulation. Second, given that *Chlorella* has been used as a health food or functional food in USA, Japan, and other countries, its safety after administration must be promising. However, the maximum sorption capacity of *Chlorella* to strontium is not very high (9.06 mg/g). In contrast, it has been reported that the maximum sorption capacity of Prussian blue to cesium is 16.5 mg/g in synthetic intestinal fluid pH 8.6 [26], 238 mg/g at pH 7.5 [27], and 715 mg/g at pH 7.5 [28]. Finally, the binding selectivity of *Chlorella* to strontium is not clear. In *in vitro* experiments, only strontium and some endogenous metals in *Chlorella* exist in the experimental system. However, because many types of metals exist under *in vivo* circumstances, the effects of some cations (Na^+^, K^+^, or Ca^2+^) on *in vitro* adsorption of ^85^Sr to *Chlorella* were evaluated (Fig 4). As the presence of the cations, especially Ca^2+^, decreased adsorption of ^85^Sr to *Chlorella* probably because of the chemical similarity between Sr and Ca, some metals, such as calcium, must compete with strontium for the *Chlorella* adsorption sites.

In summary, *Chlorella* did not adsorb cesium at any pH range but adsorbed strontium under neutral and weak basic pH conditions in *in vitro* experiments. Bone accumulation of ^85^Sr in the *Chlorella* pretreatment group was significantly lower than that in the non-treatment control group. Therefore, these results indicated that *Chlorella* could inhibit strontium absorption and enhance strontium elimination from the body through adsorption between *Chlorella* and strontium in the intestine. Further studies are required to elucidate the mechanism and the components of *Chlorella* needed for adsorption to strontium and could promote the development of more effective decorporation agents.
